# Water-Dispersible Palmitoylethanolamide (PEA) Supplementation for Functional Improvement in Adults With Chronic Sciatica-Related Back Pain: A Pilot Clinical Trial

**DOI:** 10.7759/cureus.108319

**Published:** 2026-05-05

**Authors:** Harshith N Raju, Deepa Subramanian, Rosario Russo

**Affiliations:** 1 Orthopaedics, Rajalakshmi Hospital and Research Center, Bengaluru, IND; 2 Family Medicine, Syncretic Clinical Research Services Pvt Ltd, Bengaluru, IND; 3 Health Sciences, Giellepi S.p.A., Seregno, ITA

**Keywords:** acmella oleracea, low back pain, neuropathic pain, nrs, palmitoylethanolamide, pea, sciatica

## Abstract

Background

Chronic pain, including low back pain (LBP), represents a major global public health issue and significantly affects quality of life. Low back pain is a highly prevalent condition worldwide and remains a growing public health concern.​​​​​​ Current conventional management includes pharmacological approaches such as nonsteroidal anti-inflammatory drugs (NSAIDs), opioids, and muscle relaxants, as well as non-pharmacological interventions including physical therapy and physiotherapy. Nutraceuticals are increasingly used as an adjunctive strategy, particularly in patients intolerant to conventional pharmacological treatments or requiring long-term management. Although some evidence supports their potential benefit, the overall quality of clinical data remains heterogeneous, and further well-designed studies are needed to establish efficacy and optimal use. Among these, palmitoylethanolamide (PEA) and botanical extracts such as *Acmella oleracea* have been individually investigated; however, evidence regarding their combined use remains limited and insufficiently explored.

Methods

This pilot, open-label clinical trial evaluated the effects of a novel nutraceutical combination containing PEA and *Acmella oleracea* extract in subjects with low back pain with sciatica over a 4-week period. Pain intensity was assessed using the Numeric Rating Scale (NRS), while pain characteristics were evaluated using the validated Douleur Neuropathique 4 (DN4) questionnaire. The impact on daily living activities, as well as safety and tolerability, was also investigated.

Results

Sixty participants were recruited. Baseline NRS score was 5.12±0.79 and showed a significant reduction as early as 3 days after supplementation, with continued improvement throughout the study period, reaching 1.65 ± 0.66 at the end of the study (p<0.001). DN4 scores also decreased significantly over time, with reductions observed across most neuropathic pain descriptors. Improvements in daily functioning were similarly significant. The nutraceutical combination was well tolerated, with no relevant adverse events reported.

Conclusions

Although preliminary, these findings suggest that the investigated nutraceutical combination may represent a safe and potentially effective approach with a relatively rapid onset of action for the management of chronic low back pain with neuropathic features, including sciatica. Further randomized, controlled studies are warranted to confirm these results and to better define the clinical role of this approach.

## Introduction

According to the International Association for the Study of Pain (IASP), pain is defined as “an unpleasant sensory and emotional experience associated with, or resembling that associated with, actual or potential tissue damage” [1]. Chronic pain is generally described as pain that persists or recurs for longer than three months and may continue even after resolution of the initial causative event. Beyond its sensory dimension, chronic pain exerts profound psychological, functional, and socioeconomic consequences, substantially impairing quality of life and representing a major public health challenge worldwide [2].

Among chronic pain conditions, low back pain (LBP) is the leading cause of disability globally. Data from the World Health Organization (WHO) report that LBP affected approximately 619 million individuals worldwide in 2020, with projections reaching 843 million cases by 2050, mainly driven by population growth and aging [3]. LBP is defined as pain localized between the lower edge of the ribs and the inferior gluteal fold, with or without radiation to one or both lower limbs (sciatica). Based on duration, LBP is categorized as acute (<6 weeks), subacute (6-12 weeks), or chronic (>12 weeks). Chronic LBP is frequently associated with persistent disability, reduced work productivity, and substantial healthcare costs. Although a specific structural cause can be identified in a minority of patients (e.g., disc herniation, spinal stenosis, intervertebral disk degeneration), up to 90% of cases are classified as non-specific LBP, where no clear cause can be determined [4]. Sciatica, characterized by radiating leg pain due to irritation or compression of lumbosacral nerve roots, represents a particularly disabling and painful subtype, often associated with neuropathic components and neuroinflammatory mechanisms.

The pathophysiology of chronic LBP with sciatica is multifactorial, involving complex interactions among persistent nociceptive stimulation, neuroinflammation, and peripheral sensitization. These processes may lead to the increased expression of inflammatory mediators, including cytokines (e.g., tumor necrosis factor (TNF)-α, IL-1β, IL-6), prostaglandins, and nitric oxide (NO), as well as the activation of intracellular signaling pathways such as nuclear factor kappa B (NF-κB) and mitogen-activated protein kinase (MAPK), which contribute to the persistence of the chronic pain state [5,6].

Current management of chronic LBP depends on the underlying cause of pain. In case of specific LBP with an identifiable etiology, treatment is primarily aimed at addressing the underlying condition. On the other hand, non-specific LBP requires a multidisciplinary approach, which combines lifestyle changes, physical therapy and exercise, psychological support, and pharmacological interventions. However, conventional pharmacotherapies, including nonsteroidal anti-inflammatory drugs (NSAIDs), opioids, muscle relaxants, and anticonvulsants, are often limited by variable efficacy, tolerability issues, and safety concerns, particularly with long-term use [7]. Consequently, there is growing interest in alternative approaches, such as nutraceutical supplementation with favorable safety profiles that may target multiple mechanisms, especially in subjects with chronic symptoms.

Palmitoylethanolamide (PEA) is an endogenous fatty acid ethanolamide belonging to the N-acylethanolamine (NAE) family, produced on demand in response to cellular stress and tissue injury. PEA is also present in various food sources, including egg yolk, soy oil, and certain vegetables such as tomatoes, as well as in legumes, including beans [8].

Palmitoylethanolamide has been extensively investigated for its anti-inflammatory, analgesic, and neuroprotective properties [9-11]. PEA primarily exerts its effects through activation of peroxisome proliferator-activated receptor alpha (PPAR-α), a nuclear receptor involved in the transcriptional regulation of inflammatory and metabolic genes [12]. Activation of PPAR-α results in downregulation of pro-inflammatory mediators, inhibition of NF-κB signaling, and reduction of cytokine release. In addition, PEA may modulate mast cell degranulation and attenuate microglial activation, thereby reducing neuroinflammation, a key driver of neuropathic and radicular pain [9]. Although PEA does not directly bind with high affinity to classical cannabinoid receptors (CB1 and CB2), it is known to exert an “entourage effect,” enhancing endocannabinoid signaling (e.g., anandamide) and indirectly influencing cannabinoid receptor activity. PEA has also been shown to interact with transient receptor potential vanilloid type 1 (TRPV1) channels, further contributing to the modulation of nociceptive transmission [12].

*Acmella oleracea* (L.) R.K. Jansen is a plant belonging to the *Asteraceae *family, traditionally used in South America, Africa, and Asia for the relief of dental and orofacial pain [13]. Its principal bioactive compound, spilanthol (an N-alkylamide), is responsible for its characteristic tingling and pungent properties. Spilanthol has demonstrated multiple biological activities relevant to pain modulation. Preclinical evidence suggests that it may inhibit nociceptive transmission through modulation of voltage-gated ion channels and interaction with cannabinoid-related pathways, likely due to structural similarities with endocannabinoid ligands [14]. Additionally, spilanthol has been described to modulate the expression of inflammatory mediators, including nitric oxide, prostaglandins (i.e., prostaglandin E2 (PGE2)), and pro-inflammatory cytokines, partly via inhibition of NF-κB and MAPK signaling pathways [15]. Furthermore, spilanthol may influence peripheral sensory nerve endings, thus producing rapid sensory effects and potentially contributing to a faster onset of action compared to compounds acting predominantly through genomic mechanisms. This multimodal activity, combining peripheral neuromodulation and anti-inflammatory effects, makes *A. oleracea *extract an attractive candidate for complementary pain management strategies.

Given the multifactorial nature of chronic LBP with sciatica, in which both inflammatory and neuropathic mechanisms may coexist, a combination approach targeting distinct yet complementary molecular pathways may provide enhanced clinical benefit. Indeed, PEA may primarily modulate neuroinflammation and glial activation through PPAR-α mediated genomic effects, whereas spilanthol may exert more rapid peripheral neuromodulatory and anti-inflammatory actions.

Considering this, the rationale for the present study is based on the potential complementary mechanisms of action of PEA and *A. oleracea* extract, which may target distinct pathways involved in pain modulation. The working hypothesis is that their combination may result in more rapid and enhanced clinical benefits compared to PEA alone.

The present open-label, prospective pilot clinical study was therefore designed as a feasibility trial to evaluate for the first time the effectiveness and tolerability of a novel, patented nutraceutical combination containing PEA and *A. oleracea* extract in adult subjects affected by chronic low back pain with sciatica persisting for more than three months. The primary objective was to assess the reduction in pain intensity by evaluating the proportion of subjects achieving a clinically meaningful reduction from moderate pain (4 < NRS < 7) to mild pain (NRS < 4) in at least 50% of participants by the end of the study. The secondary outcomes included longitudinal changes in pain intensity over time, changes in neuropathic features, and product tolerability. Lastly, we assessed the effect of the investigational supplement on several daily functions as an exploratory outcome.

## Materials and methods

Study design and participants

The present trial was designed as an open-label, single-center, prospective clinical study. It was carried out in accordance with the Declaration of Helsinki and Good Clinical Practice and approved by the Institutional Ethics Committee of the Rajalakshmi Hospital (protocol code GLI_SCRS_PEA_2025, approved on July 10^th^ 2025). The study was prospectively registered in the Clinical Trials Registry of India (CTRI) with the identification number CTRI/2025/08/092460. 

Adult subjects aged 18-65 years, both male and female, diagnosed with moderate low back pain (4<NRS<6) associated with sciatica for at least 3 months, were recruited. Inclusion and exclusion criteria are shown in Table 1. Written informed consent was obtained from all participants before enrollment.

**Table 1:** Inclusion and exlusion criteria NRS: numeric rating scale; DN4: Douleur Neuropathique en 4 Questions; OCPs: oral contraceptive pills; PEA: palmitoylethanolamide; LBP: low back pain; NSAIDs: nonsteroidal anti-inflammatory drugs.

Inclusion criteria	Exclusion criteria
Adult male or female subjects (age 18-65 yrs).	Any serious spinal pathology (e.g., cauda equina syndrome, spinal fracture).
Subjects diagnosed with chronic lower back pain due to sciatica, lasting for at least 3 months.	Spinal surgery or interventional procedures scheduled for sciatica during the 4-week treatment period.
Moderate pain (4 < NRS < 6).	Any treatment for neuropathic pain, tricyclic antidepressant, or sedative drugs.
DN4 score ≥ 4.	Hormonal therapy and OCPs
Written informed consent.	Any nutraceutical supplementation with nutraceuticals containing PEA, alpha lipoic acids, vitamin D, calcium, botanical extracts, and other functional ingredients able to reduce LBP and/or interfere with the endocannabinoid system.
Ability to comply with study procedures.	Steroidal anti-inflammatory drugs, NSAIDs during the month prior to screening.
	Any fractures, muscle sprains, as well as concomitant diseases (i.e., cancer, kidney diseases, liver diseases, neurological diseases, diabetes, gastritis, psychiatric diseases, blood disorders, gastrointestinal diseases, infections).
	Any alcohol or drug addiction.
	Subjects with known sensitivity to any components of the investigational product.
	Pregnant or breastfeeding women.

Investigational product and administration

The investigational product has been developed by Giellepi S.p.A. (Seregno, Italy) and is marketed as Wellpea®. It consists of a nutraceutical combination comprising palmitoylethanolamide (PEA, 90%), *Acmella oleracea* flower extract (1.5%), and functional excipients (8.5%), which are used to enhance wettability and dispersion.

The formulation is patented (WO2026027997A1) and incorporates functional excipients to enhance powder wettability, thereby improving dispersibility and dissolution. The final product is a water-dispersible powder suitable for use as a functional ingredient in food supplements and nutraceutical formulations.

At the baseline visit (Day 0), each participant received the investigational product and was instructed to take one sachet of Wellpea® (670 mg/day) dissolved in a glass of water once daily, in the morning after breakfast, throughout the study period.

Study outcomes and procedures

The primary outcome was the proportion of participants achieving a clinically meaningful reduction of pain intensity, defined as NRS<4 in at least 50% of subjects by the end of the study.

Secondary outcomes included: longitudinal changes in pain severity throughout the study period, assessed using the NRS; changes in DN4 score; and safety and tolerability. Improvements in the ability to perform daily activities affected by LBP were included as an exploratory outcome; this was considered sufficient to obtain preliminary real-world evidence on symptom perception and subject-reported outcomes within an exploratory setting.

Pain intensity was assessed using the NRS, a validated 11-point self-reported scale ranging from 0 (no pain) to 10 (worst imaginable pain). Pain severity is commonly categorized as no pain (score 0), mild (score 1-3), moderate (score 4-6), and severe (score 7-10) pain [16]. NRS scores were recorded daily by participants throughout the study period. For descriptive purposes, weekly averages were calculated and are presented in tables and figures. For inferential analyses, the full set of daily observations was used.

Neuropathic pain was evaluated using the DN4 questionnaire, a validated tool administered by the principal investigator (PI). The DN4 consists of 10 items, seven related to symptoms and three based on clinical examination. A total score ≥ 4 is indicative of neuropathic pain [17]. DN4 was captured at baseline and at the end of the study by the principal investigator (PI).

Changes in the ability to perform daily activities were analyzed as an exploratory outcome using structured questions administered by the PI. This assessment included five items evaluating personal care, walking, standing, sleeping, and social life. Responses were rated on a scale from 0 (no impairment) to 5 (complete disability). The items used in the present study are listed in Appendix 1.

This assessment was performed at each scheduled visit from baseline to the end of the study.

The Patient Global Impression of Change (PGIC) scale was used to evaluate patient-reported global improvement and tolerability using a five-point ordinal scale (Very much improved, Improved, No change, Worse, Very much worse). PGIC scores were recorded at baseline and at the end of the study and summarized descriptively as percentages. For clinical interpretation, a responder analysis was performed, defining responders as subjects reporting “Very much improved” or “Improved”.

Safety assessment

Safety was monitored throughout the study period by recording adverse events (AE), performing clinical examination, and assessing vital signs at each visit.

Hematological and biochemical parameters were evaluated using blood samples collected from each participant at baseline and at the end of the study, and were used for safety assessment. Laboratory evaluations included complete white blood cell (WBC) count with differential (neutrophils, eosinophils, basophils, monocytes, lymphocytes), red blood cell (RBC) count, packed cell volume (PCV), platelet count, hemoglobin, serum creatinine, alanine aminotransferase (ALT), and aspartate aminotransferase (AST).

Statistical analysis

This trial was designed as a pilot study; therefore, no formal sample size calculation was performed. Nevertheless, the sample size was considered adequate for the exploratory nature of the investigation. 

All statistical analyses were performed using SAS software version 9.4 (SAS Institute, Cary, USA). All enrolled participants completed the study and were included in the analysis.

The primary endpoint was the proportion of participants achieving an NRS score < 4 at week 4. The observed proportion was compared with the predefined threshold of 50% using a one-sample exact binomial test. Exact 95% confidence intervals were calculated using the Clopper-Pearson method.

Changes in Numeric Rating Scale (NRS) scores over time were analyzed using Linear Mixed-effects Models (LMMs). Time (days since baseline) was included as a continuous fixed effect, while subject was modeled as a random intercept to account for within-subject correlations. For descriptive purposes, results are also presented at weekly time points (week 1-4). Weekly mean NRS values were calculated as the average of the 7 daily scores preceding each visit, and ΔNRS was defined as the difference between these weekly means and baseline. Post hoc comparisons between baseline and each subsequent time point were performed using Holm-Bonferroni correction for multiple comparisons.

Changes in DN4 total score between baseline and week 4 were assessed using the Wilcoxon signed-rank test for paired data. Results are presented as median and interquartile range (IQR, Q1-Q3). Changes in the proportion of subjects reporting each DN4 item between baseline and week 4 were assessed using McNemar’s test for paired binary data.

Items of the daily life functionality questionnaire were analyzed both individually and as a composite total score (Appendix 1). Each item was treated as an ordinal variable (0-5 scale), and the total score (TS) was calculated as the sum of the five domains (range 0-25). Changes over time were assessed using the Friedman test for repeated measures. When significant, post-hoc pairwise comparisons were performed using the Wilcoxon signed-rank test with Bonferroni correction. The effect size for the Friedman test was calculated using Kendall’s W coefficient.

All results are presented as mean ± SD, unless otherwise specified. A two-sided p-value < 0.05 was considered statistically significant.

## Results

Participants’ characteristics

**Figure 1:**
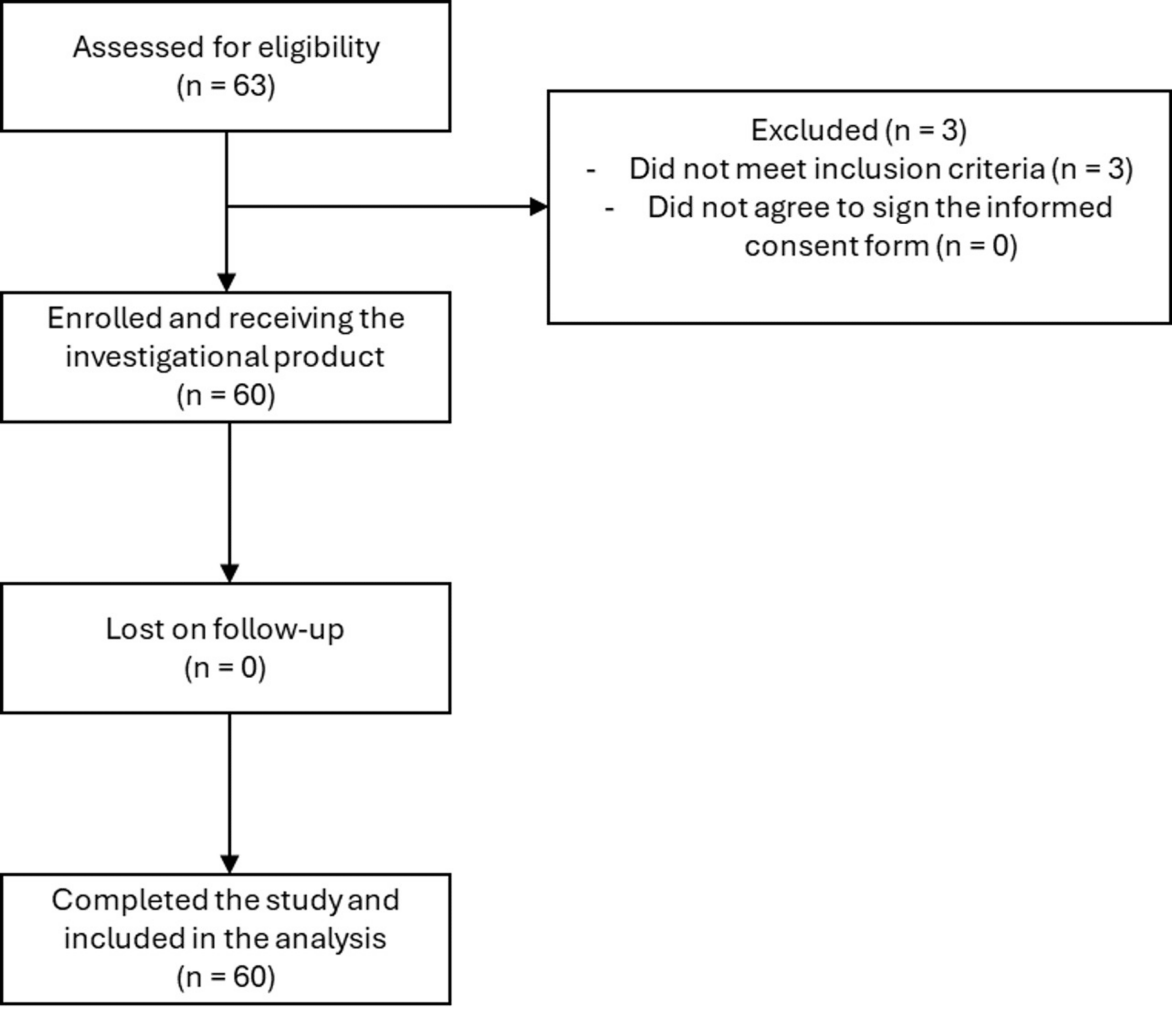
Flow chart diagram of the trial.

Sixty-three subjects were assessed for eligibility, and 60 met the inclusion and exclusion criteria (Table 1) and were subsequently enrolled in the trial. Figure 1 shows the study flow chart.

Among the 60 enrolled subjects, 28 were female, and 32 were male. The mean age was 46.6 (± 8.21) years. At baseline, none of the participants were receiving pharmacological treatment. Baseline characteristics are summarized in Table 2. All participants completed the trial; no protocol deviations or adverse events were recorded throughout the study period. Compliance was 100% at study completion.

**Table 2:** Demographic characteristics at baseline. BMI: body mass index; NRS: numeric rating scale; DN4: Douleur Neuropathique en 4 Questions.

Parameter	Value (mean±S.D.)
Age (years)	46.6 ± 8.21
Gender (M/F)	32/28
BMI (kg/m^2^)	25.64 ± 2.51
NRS (score)	5.12 ± 0.79
DN4 (score)	6.0 ± 0.74

Primary outcome: effect of supplementation on low back pain

**Figure 2:**
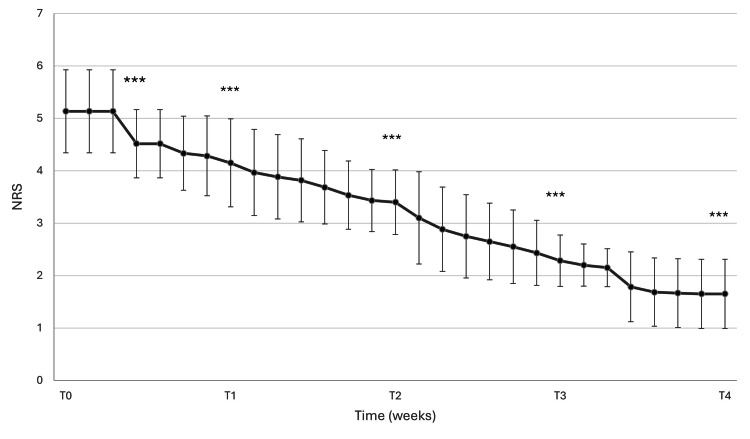
NRS following the supplementation, with the IP from baseline (T0) to the end of the study (T4). The data are shown as mean values ± SD at each visit. *** p < 0.001 vs baseline (T0) with Holm–Bonferroni correction. NRS: numeric rating scale; IP: investigational product

A significant reduction in NRS scores over time was recorded (β = -0.133 per day, 95% CI -0.135 to -0.131, p < 0.001). The mean NRS score at baseline was 5.12 (95% CI 4.97-5.27), with a progressive decrease over the 28-day follow-up period, corresponding to an overall reduction of 3.47 points. The observed mean NRS score at baseline was 5.12 ± 0.79, consistent with moderate pain intensity. As early as 3 days after the beginning of the supplementation, a significant 11.9% reduction was observed. NRS scores continued to decrease throughout the study period, reaching a mean value of 4.15 ± 0.84 at week 1, 3.40 ± 0.62 at week 2, 2.28 ± 0.49 at week 3, and 1.65 ± 0.66 at the end of the study (week 4), as shown in Figure 2.

At week 4, all participants (60/60; 100%) achieved an NRS score < 4. The responder rate was statistically significant (one-sample exact binomial test, p < 0.001), with a 95% confidence interval of 94.0%-100%. The proportion of participants achieving an NRS score < 4 increased progressively over time. Notably, after 12 days of supplementation with the investigational product, 55% of participants transitioned from moderate pain (NRS > 4) to mild pain (NRS < 4) with a mean NRS score of 3.53 ± 0.65.

Although descriptive summaries are presented at weekly intervals, the statistical model incorporates daily measurements.

Overall, these findings indicate a rapid and clinically relevant reduction in pain intensity associated with the investigated supplement, with most participants reporting a shift toward mild perception. The magnitude of the reduction corresponded to a 64% decrease in mean pain intensity over the 4-week period.

All post hoc comparisons between baseline and subsequent time points remained statistically significant after Holm-Bonferroni correction (adjusted p < 0.001). 

Table 3 shows ΔNRS vs baseline over a 4-week study period. Mean changes (Δ) were calculated as the difference between the weekly observed mean NRS score and the baseline value and presented with corresponding 95% confidence intervals.

**Table 3:** Main decreases in NRS scores over time. NRS: numeric rating scale; CI: confidence interval; ΔNRS: difference between weekly means and baseline

Timepoint	ΔNRS vs Baseline	95% CI	p-value
Week 1	−0.55	−0.65 to −0.45	<0.001
Week 2	−1.46	−1.52 to −1.40	<0.001
Week 3	−2.47	−2.54 to −2.40	<0.001
Week 4	−3.31	−3.36 to −3.24	<0.001

Secondary outcomes: DN4

**Figure 3:**
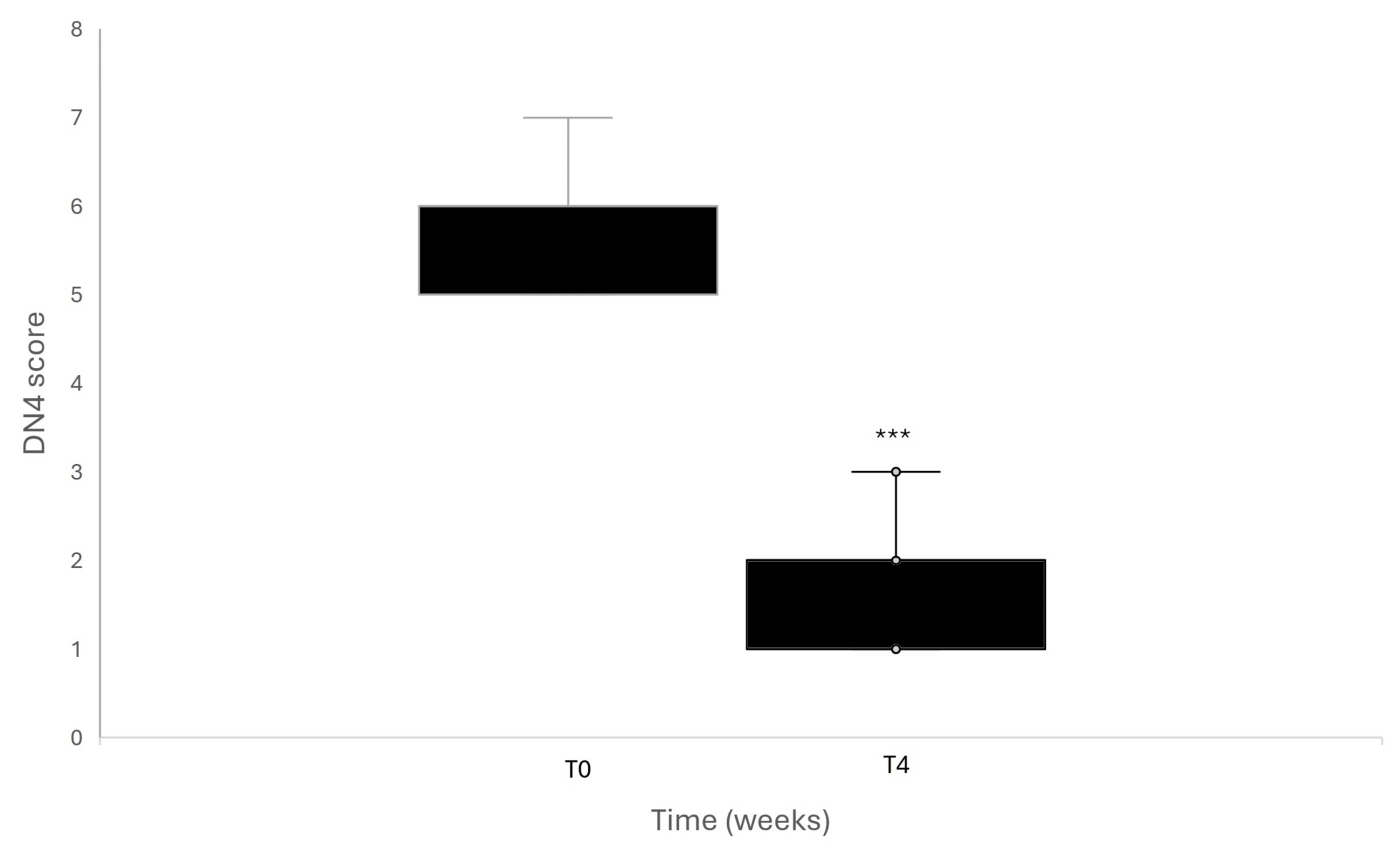
Douleur Neuropathique 4 (DN4) scores. Results are shown as boxplots with median and interquartile range, with whiskers indicating data dispersion and individual outliers. ***p<0.001 vs baseline. The Wilcoxon signed-rank test was used for statistical analysis.

Douleur Neuropathique 4 (DN4) was used to assess the pain characteristics, including both sensory descriptors and signs related to sensory examination [18]. The total DN4 score significantly decreased from baseline to week 4 (Figure 3). The median DN4 total score decreased from 6.0 (5.0-6.0) at baseline to 1.0 (1.0-2.0) at week 4. This reduction was highly statistically significant (Wilcoxon signed-rank test, p < 0.001), indicating a marked improvement in neuropathic pain features over the course of the study. The magnitude of the effect was large (r = 0.87).

At screening, the majority of subjects reported symptoms consistent with neuropathic pain according to the DN4 questionnaire. A significant reduction over time was observed across most neuropathic pain descriptors.

A burning sensation was present in 58/60 subjects (96.7%) at baseline, with resolution reported in 22 subjects (37.9%) at week 4, while 36 subjects (62.1%) remained symptomatic (p < 0.001). Painful cold sensation was reported by 54 subjects (90.0%) at baseline; by week 4, 45 subjects (83.3%) experienced resolution, whereas 9 subjects (16.7%) remained symptomatic (p < 0.001).

Electric shock-like pain was reported by three subjects (5.0%) at screening and persisted unchanged at week 4 in all cases (100%), with no significant variation over time (p = 1.000). Tingling sensation was present in 53 subjects (88.3%) at baseline, with resolution observed in 22 subjects (41.5%) at week 4, while 31 subjects (58.5%) reported persistent symptoms (p < 0.001).

Similarly, pain described as pins and needles was reported by 58 subjects (96.7%) at screening, with 39 subjects (67.2%) experiencing resolution at the end of the study and 19 subjects (32.8%) reporting persistence (p < 0.001). Numbness was reported by 58 subjects (96.7%) at baseline; of these, 22 subjects (37.9%) showed resolution at week 4, while 36 subjects (62.1%) remained symptomatic (p < 0.001).

Itching was less frequently reported, occurring in 11 subjects (18.3%) at screening; of these, 10 subjects (90.9%) experienced resolution, while one subject (9.1%) reported persistent symptoms at week 4 (p = 0.002). No subjects reported hypoesthesia to touch or pinprick at either baseline or at week 4 (0%). Brushing-evoked pain was reported by 58 subjects (96.7%) at screening, with 42 subjects (72.4%) reporting resolution by the end of the study, while 16 subjects (27.6%) continued to experience this symptom (p < 0.001).

**Figure 4:**
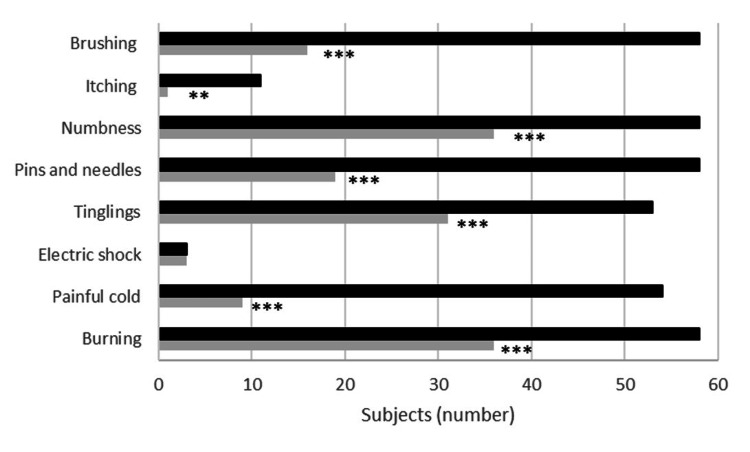
Responder analysis presented as stacked bars. The DN4 score for each sign assessed at the end of supplementation. Black: number of subjects with symptoms at baseline; grey: number of subjects with symptoms at the end of supplementation. **p<0.01 vs baseline; ***p<0.001 vs baseline. The McNemar test was used for statistical analysis. DN4: Douleur Neuropathique 4 questionnaire

Overall, the highest resolution rates were observed for painful cold sensation and brushing-evoked pain, whereas no improvement was detected for electric shock-like pain. Changes in the presence of individual DN4 symptoms between baseline and week 4 were evaluated using McNemar’s test. The results are summarized in Figure 4.

Secondary outcomes: daily life functionality questionnaire

**Figure 5:**
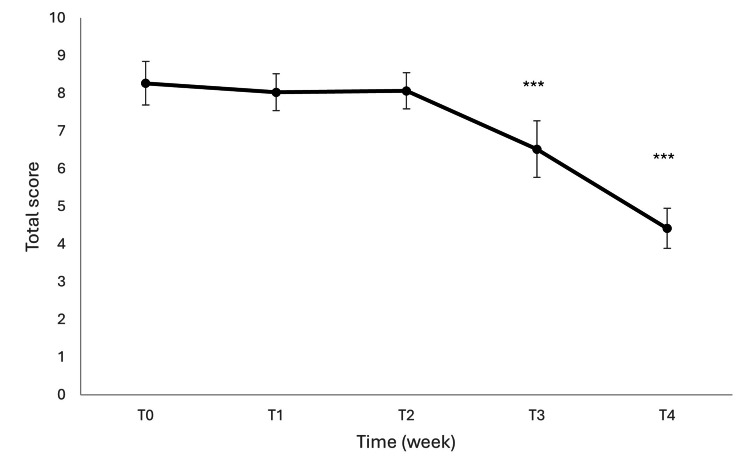
Changes in total daily life functionality score over time. Data are presented as mean ± SD. A significant reduction in disability was observed from week 3 onwards (Friedman test, ***p < 0.001).

Exploratory analyses of the investigator-reported questionnaire showed a progressive improvement across all functional domains over time. The impact of treatment on daily life functions was assessed using a non-validated, investigator-administered questionnaire evaluating five domains (personal care, walking, standing, sleeping, and social life) on a 0-5 ordinal scale. A total score (range 0-25) was calculated as the sum of all items. A significant improvement over time was observed in the total score (Friedman test, χ² = 217.98, p < 0.001). Mean scores decreased progressively from baseline (8.27 ± 0.58) to week 1 (8.03 ± 0.49) and week 2 (8.07 ± 0.48), with a more pronounced reduction at week 3 (6.52 ± 0.75) and week 4 (4.42 ± 0.53). The results are shown in Figure 5.

The impact of treatment on daily life functions was further explored at the item level. All five domains showed a significant change over time (Friedman test, all p < 0.001). In particular, walking and standing exhibited the highest baseline impairment and the most pronounced improvement over time, while sleeping and personal care showed more moderate but consistent reductions in scores. Social life demonstrated a progressive improvement, especially between week 3 and week 4. Post-hoc pairwise comparisons (Wilcoxon signed-rank test with Bonferroni correction) revealed that the most significant differences occurred between baseline and week 3, and between baseline and week 4 across all domains (all adjusted p < 0.001).

The overall effect size was large (Kendall’s W = 0.73), indicating a strong treatment effect on functional disability over time.

These findings indicate a clinically meaningful improvement in functional disability, particularly from the third week of supplementation onward.

Secondary outcomes: Patients' Global Impression of Change (PGIC)

At week 4, 51.7% of subjects reported a “very much improved” condition, while the remaining 48.3% reported “improved” symptoms. No cases of worsening were observed. For clinical interpretation, a responder analysis was performed, defining responders as subjects reporting “very much improved” or “improved”. According to this criterion, the responder rate was 100%.

The investigational product was well tolerated, with no adverse events reported throughout the study period. All enrolled subjects completed the study, and no rescue medication was required. No clinically relevant changes in physical examination findings or vital signs were observed throughout the study period. Similarly, no significant changes were detected in hematological or biochemical parameters at the end of supplementation compared with baseline (the results are shown in Appendix 2). 

## Discussion

The present trial investigated the clinical effectiveness of a novel patented nutraceutical functional ingredient containing palmitoylethanolamide (PEA) and an extract of the aerial parts of *Acmella oleracea* in subjects with low back pain and sciatica. This combination was developed to target both central and peripheral pain pathways, potentially providing complementary effects.

Palmitoylethanolamide is an endogenous fatty acid amide belonging to the N-acylethanolamine family, widely recognized for its role in modulating neuroinflammation and pain signaling [10,11]. Its primary mechanism of action involves the activation of peroxisome proliferator-activated receptor alpha (PPAR-α), leading to the downregulation of pro-inflammatory mediators such as TNF-α, IL-1β, and cyclooxygenase-2 (COX-2) [19]. In addition, PEA exerts an “ALIA” (autacoid local injury antagonism) effect by modulating mast cell degranulation and microglial activation, which are key contributors to peripheral and central sensitization. Furthermore, PEA indirectly enhances endocannabinoid signaling (the so-called “entourage effect”) by inhibiting the degradation of anandamide, thereby contributing to analgesic and anti-inflammatory responses.

From a molecular standpoint, PEA has also been shown to reduce oxidative stress by improving mitochondrial function and decreasing reactive oxygen species (ROS) production. It may also modulate NF-κB signaling, thereby contributing to a broader anti-inflammatory transcriptional profile and helping to restore cellular homeostasis under conditions of chronic inflammation [20].

On the other hand, *Acmella oleracea* extract appears to act primarily at the peripheral level, largely due to the presence of alkylamides such as spilanthol. This bioactive N-alkylamide, structurally similar to endocannabinoids, is capable of interacting with cannabinoid receptors (CB1 and CB2) as well as transient receptor potential (TRP) channels, including TRPV1 and transient receptor potential ankyrin 1 (TRPA1), which are critically involved in nociceptive transmission. These interactions may contribute to its relatively rapid analgesic effects [14].

In addition, spilanthol has been shown to inhibit key inflammatory pathways, including NF-κB and MAPK signaling cascades, resulting in reduced production of nitric oxide (NO), prostaglandins (e.g., PGE2), and pro-inflammatory cytokines [15,21]. It has also been reported to activate the nuclear factor erythroid 2-related factor 2 (Nrf2)pathway, promoting the expression of endogenous antioxidant enzymes such as superoxide dismutase (SOD), catalase, and glutathione peroxidase, thereby contributing to cytoprotection and improved tissue resilience [20].

Taken together, PEA and *A. oleracea* extract may provide a dual mechanism of action, combining rapid peripheral modulation of nociceptive signaling with longer-lasting effects on neuroinflammation and pain sensitization. This integrated approach may be particularly relevant in conditions such as low back pain with sciatica, where both inflammatory and neuropathic components coexist.

Inflammation, peripheral sensitization, and structural changes in spinal innervation are well-documented features in individuals with back pain, as well as in preclinical models [22]. Even in the absence of overt nerve compression following intervertebral disc herniation, biochemical and neuroplastic changes may occur within the peripheral nervous system, contributing to pain generation and chronification. Clinical studies have demonstrated elevated levels of inflammatory mediators in serum and affected tissues of patients with low back pain [23,24] while animal models have shown that disc degeneration is associated with increased inflammatory signaling, enhanced sensory innervation, and plastic changes in both peripheral and central neurons [25,26]. Overall, these findings provide a biologically plausible framework supporting the observed therapeutic effects of the investigated nutraceutical combination.

The results of the present study demonstrate a significant and progressive reduction in pain intensity, as measured by the NRS, over a 28-day supplementation period in a cohort of 60 subjects. Notably, a statistically significant decrease in NRS scores was observed as early as the first week, with some subjects reporting improvement within 3 days. This early change was derived from the analysis of daily NRS recordings. This early onset of action was followed by a consistent and clinically meaningful reduction over time, reaching an overall change from baseline (ΔNRS) of -3.31. From a clinical standpoint, a reduction of ≥2 points on the NRS is generally considered meaningful in pain research, supporting the relevance of the changes observed in this study [27]. The progressive nature of this response may suggest a cumulative effect, potentially driven by the sustained anti-inflammatory and neuroprotective activity of PEA.

The rapid symptom relief observed is particularly relevant in a clinical context and may translate into improved symptom control, enhanced patient adherence, greater perceived treatment efficacy, and improved overall quality of life. Early improvements have been associated with more favorable long-term outcomes in pain management [28].

Importantly, the neuropathic component of pain, as assessed by the DN4 questionnaire, appears to be consistent with the proposed mechanism of action of the investigational product. The average reduction in DN4 scores recorded in the present study (from 5.72 ± 0.74 at baseline to 1.62 ± 0.67 at week 4) is consistent with the hypothesis that the intervention may modulate both nociceptive and neuropathic pain components, possibly through combined effects on peripheral nociceptors, microglial activation, and endocannabinoid signaling.

The investigational supplement was also associated with improvements in activities of daily living, as observed over the course of the study. Although the questionnaire used in this study was not formally validated, its longitudinal consistency may suggest that it was sufficiently sensitive to detect clinically meaningful changes over time. These findings should nevertheless be interpreted with caution and confirmed using validated instruments in future studies.

From a clinical perspective, these findings may have several practical implications. The rapid and clinically meaningful reduction in pain intensity, together with the improvement in neuropathic features, may support the potential role of this formulation as an adjunctive strategy in the management of chronic low back pain, particularly in subjects with mixed nociceptive and neuropathic components. In addition, the favourable tolerability profile observed in this study suggests that this approach may be suitable for individuals who are unable to tolerate conventional pharmacological treatments or who require long-term management strategies. The possibility of achieving early symptom relief may also contribute to improving adherence and engagement in non-pharmacological interventions, such as physical therapy and structured exercise programs, which remain key elements of multidisciplinary care.

In line with existing literature, individuals with low back pain often exhibit reduced pressure pain thresholds, reflecting localized hypersensitivity and central sensitization [22]. The observed clinical improvements may therefore indicate not only symptomatic relief but also a partial normalization of pain processing mechanisms.

Previous clinical studies investigating the effects of PEA in subjects with LBP have consistently reported beneficial effects on pain intensity and functional outcomes, both as monotherapy and in combination with other complementary and alternative approaches, including structured rehabilitation and manual therapies. However, these studies are highly heterogeneous in terms of formulation, dosing regimens, co-interventions, and design, making direct comparisons challenging [29,30]. One notable difference emerging from the literature concerns the onset of the clinical effects. Clinically meaningful improvements are generally reported after several weeks of treatment, typically within observation periods ranging from 4 to 8 weeks or longer. For instance, a recent randomized, placebo-controlled trial investigating a phospholipid-based PEA formulation in patients with moderate chronic LBP with a neuropathic component demonstrated clinically meaningful reductions in pain severity and functional disability after 8 weeks of treatment, in addition to a standard of care, including commonly prescribed analgesics such as paracetamol and NSAIDs [29].

Similarly, a large observational cohort study in elderly subjects with chronic LBP reported that a combination treatment including oxygen-ozone therapy and oral PEA (600 mg/day), alpha-lipoic acid, and myrrh led to significant reductions in pain intensity at one month, which were sustained at one-year follow-up [30]. By comparison, the present study achieved a comparable magnitude of pain reduction using a standard daily dose of PEA (600 mg/day) without the need for intensive adjunctive therapies. While these findings should be interpreted with caution due to differences in study design, they may further support the potential of the investigated product as a standalone approach in the management of chronic low back pain with sciatica.

In contrast, the present study provides evidence of a markedly earlier onset of action, with approximately 50% of participants achieving mild pain levels (NRS < 4) within 12 days of supplementation. This rapid response is not commonly emphasized in previous reports and may represent a distinguishing feature of the investigated combination. Moreover, such clinical effects were observed with a daily dose of PEA (600 mg/day), which is consistent with commonly used dosages reported in the literature, including studies employing micronized or ultramicronized formulations as well as more advanced delivery systems such as phospholipid-based complexes.

Furthermore, the formulation used in the present study does not rely on micronization strategies. Nevertheless, the observed clinical outcomes may indicate that factors other than particle size, including formulation characteristics affecting dispersion and dissolution, as well as the potential synergistic interaction with complementary bioactive compounds present in the *A. oleracea* extract, such as spilanthol, may play a relevant role in determining clinical efficacy.

Taken together, these considerations suggest that the combination tested in the present study may offer a distinctive clinical profile characterized by a relatively rapid onset of action and meaningful pain reduction at a standard dosage, without the need for specialized micronization processes or complex multimodal treatment strategies. However, given the differences in study designs and the absence of direct comparisons, these observations should be interpreted with caution and warrant further investigation in appropriately designed placebo-controlled trials.

The present study has several limitations. The open-label design and the absence of a control group limit the strength of causal inference and increase the possibility of placebo effects or regression to the mean. The reliance on self-reported outcomes introduces inherent subjectivity, although this is common in pain research; this may have contributed to the very high responder rate observed on the PGIC scale and, consequently, to a potential overestimation of treatment effects. Additionally, the functional assessment tool employed was not formally validated, and the lack of long-term follow-up prevents conclusions regarding the persistence of the observed benefits after discontinuation of supplementation. Another limitation is the absence of radiological assessments, such as magnetic resonance imaging (MRI), which could have provided complementary information on structural abnormalities underlying low back pain. However, the primary aim of this exploratory study was to evaluate changes in pain intensity and patient-reported outcomes rather than to investigate structural-pathological correlations.

Finally, the assessment of psychosocial risk factors using validated tools such as the STarT Back Screening Tool (Keele Health, Staffordshire, UK) was not included in the protocol. This instrument is widely used to stratify patients with low back pain according to their risk of persistent disability and to guide stratified therapeutic approaches. However, the primary aim of the present study was not to evaluate prognostic risk or to tailor interventions based on psychosocial profiles, but rather to generate preliminary evidence on pain intensity and neuropathic features. Therefore, while the inclusion of such a tool could have provided additional insight into patient heterogeneity and potential confounding factors, its absence does not compromise the primary exploratory objectives of the study.

Despite these limitations, the use of a linear mixed-effects model represents a methodological strength, allowing appropriate handling of repeated measures and providing robust estimates of treatment effects over time. The consistency between weekly categorical analyses and continuous time modeling further strengthens the reliability of the findings.

Although the absence of a control group represents a limitation, this choice was intentional and aligned with the pilot, exploratory nature of the study, whose primary aim was to assess the feasibility and tolerability of the investigational formulation before conducting a randomized, double blind, placebo-controlled study. Open-label, uncontrolled designs are commonly employed in early-phase investigations to generate preliminary data that can inform the design, sample size estimation, and endpoint selection of subsequent controlled trials. Additionally, the pragmatic design of the study reflects a real-world clinical setting in which subjects with persistent low back pain received the nutraceutical formulation as a standalone intervention.

Finally, it is worth noting that most previous studies on PEA have employed capsule or tablet formulations. To the best of our knowledge, this is the first study evaluating this combination in a water-dispersible powder sachet formulation, which may offer potential advantages in terms of absorption kinetics, user compliance, and ease of administration. However, comparative pharmacokinetic data are required to substantiate this hypothesis.

## Conclusions

The results of this clinical study provide preliminary evidence of a potential benefit of the combination of PEA and *Acmella oleracea* extract in relieving pain in subjects with chronic low back pain with sciatica. The early onset of action, observed within a few days of supplementation, may support a potential synergistic interaction between the two functional components in mediating clinical benefits, including a fast reduction in pain intensity. In addition, the favorable safety profile of the tested combination, together with the absence of adverse events during the entire trial, supports its use in the target population. Given that chronic low back pain is a highly prevalent and disabling condition affecting millions of individuals worldwide, the tested product may represent a promising complementary approach for chronic pain management. Future blinded, randomized controlled trials are warranted to confirm these findings and to further elucidate the mechanisms underlying the observed clinical effects. Moreover, longer follow-up periods would be valuable to assess the durability and clinical relevance of the response over time.

## Appendices

Appendix 1: Functionality questionnaire

**Table 4:** Functionality questionnaire

1. Personal care	Are you able to take a shower, go in/out the bath, put on/off your socks, wear your clothes?				
No impairment at all (0)	With a little difficulty (1)	With some difficulty (2)	With difficulty (3)	With much difficulty (4)	Unable to do (5)
2. Walking	Are you able to to go for a walk, shopping, or to go up and down stairs at a normal pace?				
No impairment at all (0)	With a little difficulty (1)	With some difficulty (2)	With difficulty (3)	With much difficulty (4)	Unable to do (5)
3. Standing	Are you able to stand for at least 15 minutes without needing to sit or lean on anything?				
No impairment at all (0)	With a little difficulty (1)	With some difficulty (2)	With difficulty (3)	With much difficulty (4)	Unable to do (5)
4. Sleeping	Are you able to sleep through the night without interruptions?				
No impairment at all (0)	With a little difficulty (1)	With some difficulty (2)	With difficulty (3)	With much difficulty (4)	Unable to do (5)
5. Social life	Are you able to do your regular leisure activities with others (such as family, friends) and participate in social activities?				
No impairment at all (0)	With a little difficulty (1)	With some difficulty (2)	With difficulty (3)	With much difficulty (4)	Unable to do (5)

 Appendix 2: Hematochemical parameters

**Table 5:** Hematochemical parameters WBC: white blood cells; RBC: red blood cells; AST: aspartate aminotransferase; ALT: alanine aminotransferase.

Parameter	Baseline	Week 4	p-value
WBC (cells/µl)	7134.50 (1549.17)	6477.70 (1137.46)	>0.05
RBC (million/mm^3^)	4.39 (0.58)	4.43 (0.57)	>0.05
Haemoglobin (g/dl)	12.44 (1.33)	12.48 (1.25)	>0.05
AST (U/L)	26.60 (5.02)	27.60 (5.51)	>0.05
ALT (U/L)	23.70 (7.73)	23.50 (7.11)	>0.05
Creatinine (mg/dL)	0.79 (0.12)	0.81 (0.11)	>0.05
